# Interaction with a phage gene underlie costs of a β-lactamase

**DOI:** 10.1128/mbio.02776-23

**Published:** 2024-01-09

**Authors:** Huei-Yi Lai, Tim F. Cooper

**Affiliations:** 1 School of Natural Sciences, Massey University, Auckland, New Zealand; University of Pittsburgh School of Medicine, Pittsburgh, Pennsylvania, USA

**Keywords:** antibiotics, antibiotic resistance genes, *relA*, bacteriophage, gene interactions

## Abstract

The fitness cost of an antibiotic resistance gene (ARG) can differ across host strains, creating refuges that allow the maintenance of an ARG in the absence of direct selection for its resistance phenotype. Despite the importance of such ARG-host interactions for predicting ARG dynamics, the basis of ARG fitness costs and their variability between hosts are not well understood. We determined the genetic basis of a host-dependent cost of a β-lactamase, *bla*_TEM-116*_, that conferred a significant cost in one *Escherichia coli* strain but was close to neutral in 11 other *Escherichia* spp. strains. Selection of a *bla*_TEM-116*_-encoding plasmid in the strain in which it initially had a high cost resulted in rapid and parallel compensation for that cost through mutations in a P1-like phage gene, *relA_P1_*. When the wild-type *relA_P1_* gene was added to a strain in which it was not present and in which *bla*_TEM-116*_ was neutral, it caused the ARG to become costly. Thus, *relA_P1_* is both necessary and sufficient to explain *bla*_TEM-116*_ costs in at least some host backgrounds. To our knowledge, these findings represent the first demonstrated case of the cost of an ARG being influenced by a genetic interaction with a phage gene. The interaction between a phage gene and a plasmid-borne ARG highlights the complexity of selective forces determining the maintenance and spread of ARGs and, by extension, encoding phage and plasmids in natural bacterial communities.

**IMPORTANCE:**

Antibiotic resistance genes (ARGs) play a major role in the increasing problem of antibiotic resistance in clinically relevant bacteria. Selection of these genes occurs in the presence of antibiotics, but their eventual success also depends on the sometimes substantial costs they impose on host bacteria in antibiotic-free environments. We evolved an ARG that confers resistance to penicillin-type antibiotics in one host in which it did confer a cost and in one host in which it did not. We found that costs were rapidly and consistently reduced through parallel genetic changes in a gene encoded by a phage that was infecting the costly host. The unmutated version of this gene was sufficient to cause the ARG to confer a cost in a host in which it was originally neutral, demonstrating an antagonism between the two genetic elements and underlining the range and complexity of pressures determining ARG dynamics in natural populations.

## INTRODUCTION

The spread of antibiotic resistance in pathogenic bacteria is a growing problem for public health (1). Although increases in resistance traits are mainly attributed to selection imposed by exposure to corresponding antibiotics, other mechanisms may also contribute to the success of antibiotic resistance traits (2–4). For example, in the absence of antibiotic selection, the genetic determinants of antibiotic resistance, antibiotic resistance mutations (ARMs), and antibiotic resistance genes (ARGs) can impose a fitness cost on bacteria (5). These costs vary between strains, potentially affecting competition outcomes within otherwise isogenic communities of sensitive or resistant strains in the absence of antibiotic selection (2, 5). It is also possible that costs can be compensated, perhaps at different rates and extents in different strains (5, 6). Understanding the costs of ARGs, and their potential to change, is crucial to understanding, and eventually predicting, the ecological and evolutionary dynamics of resistance traits in bacterial populations.

Whereas ARMs generally occur in host cell genes, ARGs are disseminated widely across bacterial species so that genetic interactions that determine their fitness cost will be diverse and can have a significant influence on their selection (7). Candidate factors linked to the success of horizontally acquired ARGs include compatibility of GC content and codon usage with the host (7, 8), gene function in the context of the host’s metabolism (9), protein interactivity (10), toxicity (11), gene expression level (12), and the 5′ untranslated region (UTR) stability of mRNA (13, 14). The effect of these factors on the costs of ~200 clinically prevalent ARGs has been examined in *Escherichia coli* (15). That study found costs could sometimes be predicted. For example, the fitness cost of cell-interacting ARGs, such as efflux proteins, was positively correlated with the phylogenetic distance between the donor bacterium and *E. coli* (15). However, no such correlation was found for drug-interacting ARGs, such as drug modification enzymes, underlining the complexity of mechanisms likely to influence ARG selection in natural populations (15). Moreover, to our knowledge, no general mechanisms have been proposed to explain the differences in the cost of a single ARG over different host strains of the same bacterial species.

Detailed studies on individual ARGs have revealed diverse specific mechanisms underlying fitness costs. For example, the cost of the *tetAR* tetracycline resistance operon has been attributed to its unregulated expression in the absence of tetracycline (16, 17). Costs of the β-lactamases BlaCTX-M-15, BlaSME-1, BlaVIM-1, and BlaSPM-1 are, in part, due to their signal peptides, which probably decrease host fitness by disrupting their cell membrane (17–19). By contrast, expression of the β-lactamases BlaOXA and BlaSFO-1 is associated with changes in host cell peptidoglycan composition, suggesting the residual DD-transpeptidase activity of β-lactamases may underlie their costs (20).

Whatever the basis of the cost of a particular ARG, those costs are themselves subject to evolutionary change. Mutations that compensate for the cost of ARM-mediated resistance occur repeatedly in laboratory evolution experiments and have been identified in some clinical isolates (21–26). These compensatory mutations often occur in genes encoding products that physically interact with those of the focal ARG, implying that studying the basis of compensation to an ARG’s cost may help to predict mechanisms through which that cost occurs (27, 28). For example, studies on different types of ARG-carrying plasmids have found compensatory mutations that effect transcription and DNA structure, suggesting that these processes are a common cause of plasmid cost (21–24, 29).

Previously, we found the β-lactamase gene, *bla*_TEM-116*_, imposed a significant fitness cost of >10% in the *E. coli* strain M114 but was only slightly costly or nearly neutral in 10 other *Escherichia* spp. host strains (30). Here, we examine the potential for compensation of this cost. We find that replicate M114 populations containing a *bla*_TEM-116*_ plasmid evolved to reduce the rates of population-level plasmid loss over time. This change was due to compensatory mutations acting to reduce the fitness cost of *bla*_TEM-116*_. Genome sequencing and genetic reconstruction experiments demonstrated that compensation was due to mutations affecting a *relA* ortholog encoded by a phage-like P1 element and that this gene was sufficient to cause *bla*_TEM-116*_ to confer a cost in a strain in which it was initially neutral.

## MATERIALS AND METHODS

### Bacterial strains, plasmids, and media

*E. coli* strains M114 and REL606 have been described previously (31). Strains were propagated in Davis Mingioli medium supplemented with 250 mg/mL glucose (DM250) at 37°C for all selection and fitness estimation experiments unless noted otherwise. Lysogeny broth (LB) supplemented with ampicillin (Ap; 100 µg/mL) as appropriate to ensure plasmid selection was used for growth as part of genetic modification protocols. Media used to grow strains containing the plasmids pmFP and pmFP-*bla*_TEM-116__*_ were supplemented with 50 mg/mL kanamycin as described in the text.

The construction and features of pmFP and pmFP-*bla*_TEM-116__*_ have been described previously (30). Briefly, pmFP is a derivative of pUA66 from which the promoterless *gfp* has been removed (32, 33). The *bla*_TEM-116__*_ gene and promoter were cloned into pmFP from pTarget to produce pmFP-*bla*_TEM-116__*_ (32, 33). Sequencing of this construct found that the *bla*_TEM-116_ gene present in pTarget differed from the reference sequence (NCBI accession U36911) by a non-synonymous mutation (Q274R) and we added an asterisk to the genotype of the allele used here to indicate this.

### Selection for ARG compensation

To select for compensation of plasmid costs, we inoculated replicate cultures with colonies of relevant strains obtained by streaking from freezer stocks. Initial cultures were grown with antibiotics as appropriate to ensure plasmids were present in all cells. Cultures were propagated by daily 1:100 dilution into fresh antibiotic-free media for 61 cycles (~400 generations where generations are estimated as log_2_ of the dilution factor multiplied by the total number of transfer cycles). At the end of this time, cultures were inoculated into DM250 supplemented with kanamycin to kill any plasmid-free cells, thereby resetting plasmid-containing cells to fixation. These “reset” cultures were then inoculated into new antibiotic-free DM250 media and propagated by daily 1:100 dilution into fresh antibiotic-free media for further 21 cycles (~140 generations). Plasmid frequency was determined periodically throughout the second part of the experiment and compared to control populations started with the same plasmid-containing host strains taken directly from freezer stocks (i.e., omitting the 61-cycle initial evolution treatment). Plasmid frequency was determined by plating population samples on selective and non-selective media to determine plasmid-containing cell and total cell counts, respectively.

### Introducing mutations into *relA*_*P1*_

Genetic modification of the P1-like phage gene, *relA_P1_*, was performed using the portMAGE protocol (34). Briefly, the portMAGE2 plasmid was used to transform target cells. Transformants were maintained at 30°C to prevent the induction of the encoded *c*I857-repressed λ red recombinase enzymes. Overnight cultures of transformants were diluted 1:200 into 5 mL LB supplemented with Ap and grown for 3 hours. To induce red recombination enzymes and a transitive mutator phenotype, these cells were induced at 42°C for 15 minutes before being placed on ice for 10 minutes and then made electrocompetent by washing three times with 10 mL of ice-cold Milli-Q water. Washed cells were resuspended in 100 µL of Milli-Q water and placed on ice before mixing with an oligonucleotide (1 µM final concentration) designed to introduce focal mutations into the target cell. Oligonucleotides were designed following published guidelines (35) and are listed in Table S1. After electroporation, cells were recovered at 30°C in LB + Ap for 2 hours before either plating on LB + Ap agar plates or inoculating into 5 mL LB + Ap for use in a subsequent portMAGE cycle. Four cycles of portMAGE were performed before genotyping randomly chosen clones to test for target modifications. To cure the portMAGE2 plasmid, cells were cultured at 42°C overnight on LB agar, then restreaked to fresh LB agar and incubated at 37°C before screening to identify ampicillin-susceptible strains.

### *relA*_*P1*_ integration at *att*Tn*7* site

To integrate *relA_P1_* into REL606, the *relA_P1_* gene, including flanking sequences extending 134 bp upstream and 140 bp downstream of the open reading frame, was cloned into the mini-Tn*7* delivery vector pGRG36 (36). The resulting plasmid, pGRG36-*relA_P1_*, was used to transform REL606, and transformants were selected on LB + Ap agar at 30°C. To induce mini-Tn*7* transposition, transformants were inoculated into LB broth supplemented with 0.1% arabinose and grown overnight at 30°C. To cure cells of pGRG36, colonies were grown and restreaked on LB agar. Integration of *relA_P1_* at the *att*Tn*7* site was confirmed by PCR.

### Fitness competitions

Flow cytometry-based competition assays were used to estimate the fitness cost of a plasmid or a gene following a protocol described previously (30). Briefly, cells were inoculated from freezer stocks into DM250 supplemented with kanamycin if they carried a plasmid and incubated overnight. These cultures were diluted 1:100 into fresh antibiotic-free DM250 for two 24-hour pre-conditioning cycles. After pre-conditioning, equal volumes of competing test and reference pUA66-*P_rpsL_gfp* plasmid-containing cells were mixed and competitor proportions were determined using flow cytometry (day 0). The cell mixture was also diluted 1:100 into fresh medium and cultured overnight before again determining the proportion of both competitors (day 1). Estimates of the fitness effect of focal plasmids or ARGs were made indirectly as the difference in fitness of competitors differing by that focal genetic trait competed separately against a common pUA66-*P_rpsL_gfp* plasmid-containing reference strain. For example, the difference in fitness of a strain containing the empty pmFP vector relative to the reference strain, and the fitness of the same strain containing the pmFP*-bla*_TEM-116*_ plasmid relative to the reference strain, was used to isolate the fitness effect of the *bla*_TEM-116*_ ARG. The fitness of a test strain relative to the reference was estimated as *w* = ln(*f*^test^_final_ × 100/*f*^test^_initial_)/log(*f*^ref^_final_ × 100/*f*^ref^_initial_), where *f*^test^ and *f*^ref^ indicate the frequency of test and reference strains, respectively, and subscripts indicate the initial and final sample times. The final frequency is multiplied by 100 to account for the 100-fold growth occurring during the competition.

### Whole-genome sequencing of evolved clones

One plasmid-carrying clone was isolated from each M114 evolved line. The genomic DNA of the evolved clones was isolated using the Wizard Genomic DNA Purification Kit (Promega) and short-read sequenced using the Illumina HiSeq platform (Microbial Genomic Sequencing Center, USA). Breseq was used to identify mutations relative to the ancestral strain (37).

### Statistics

The R statistical computing platform version 4.3.1 was used for all analysis and visualization (38). The flowCore package was used to analyze flow cytometry data, and the Binom.confit function from the binom package was used to calculate confidence intervals on proportions (39).

## RESULTS

### Variation in fitness costs leads to different ecological and evolutionary dynamics of the *bla*_TEM-116*_ plasmid

Variation in the fitness cost of a given ARG across different bacterial strains is expected to affect the strength of both purifying selection and selection for compensation of costs. As a first step to test this expectation, we examined the stability of a non-conjugative plasmid, pmFP, encoding *bla*_TEM-116__*_ in two hosts, REL606 and M114, in which it confers different costs [cost in REL606: 0.3% (±1.0 95% CI); cost in M114: 10.9% (±3.3 95% CI)].

Replicate lines started with M114 carrying either pmFP-*bla*_TEM-116*_ or a pmFP vector-only control plasmid were initially propagated for 400 generations without any direct selection for the plasmids. In the M114 populations, in which the pmFP-*bla*_TEM-116*_ plasmid had a high cost, it remained present in an average of only 4.5% (0.2–11.1 95% CI) of cells at the end of the selection period. By contrast, the control plasmid remained present in 93.9% of cells (86.8–97.5 95% CI). The small number of cells retaining the pmFP-*bla*_TEM-116*_ plasmid indicates the action of strong purifying selection against plasmid carriage and, therefore, the potential for selection of compensatory mutations that reduce the cost of carriage. Indeed, by itself, purifying selection is expected to lead to plasmid carriage rates of <<0.1% after 400 generations of selection given a 10.9% cost so that observed plasmid-carriage rates above this level are consistent with compensatory mutations stabilizing the pmFP-*bla*_TEM-116*_ plasmid.

**Fig 1:**
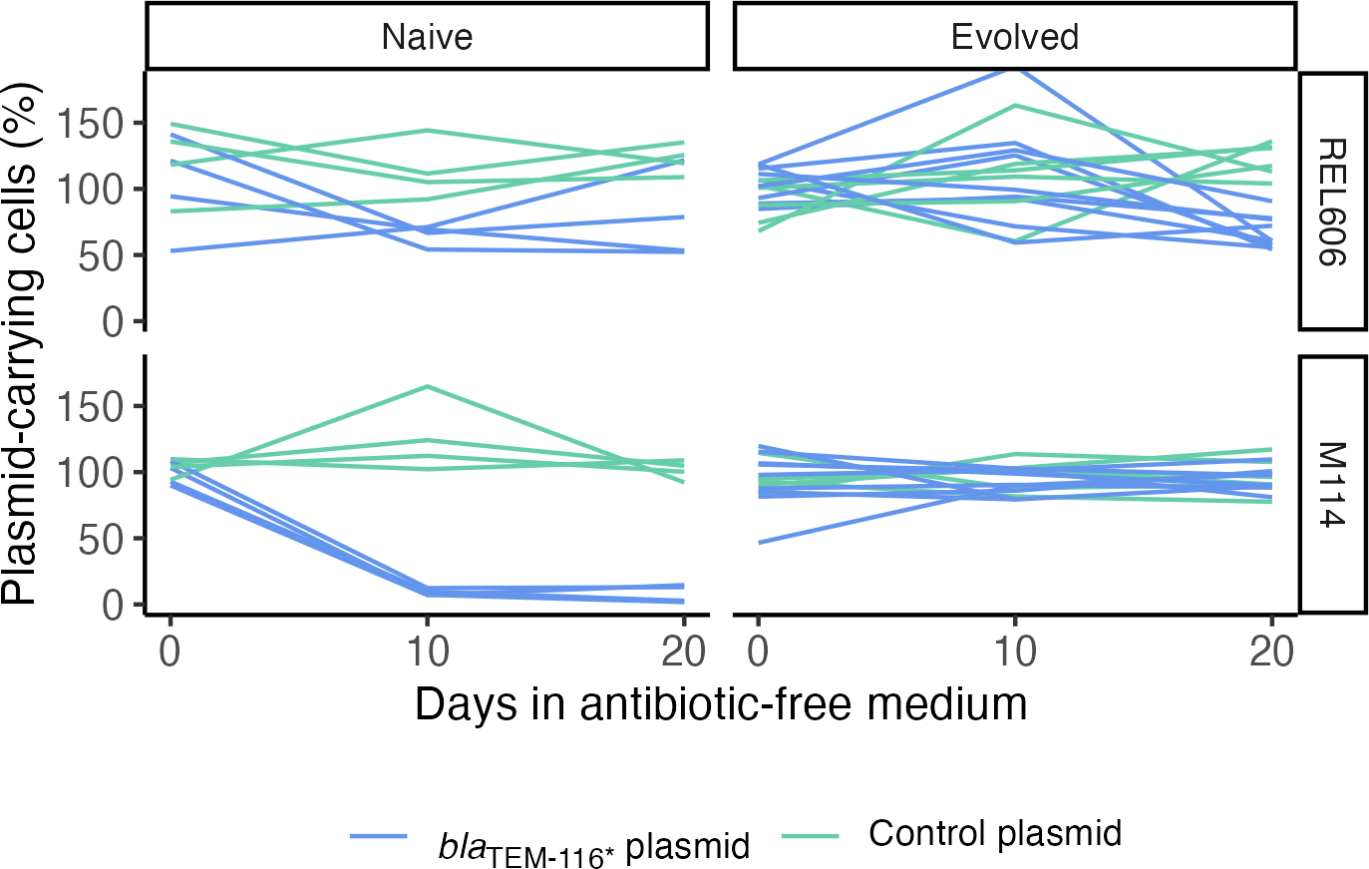
Dynamics of control and bla_TEM-116*_ plasmids in naive and pre-evolved populations. Dynamics of control (empty vector, pmFP) and pmFP-*bla*_TEM-116__*_ plasmid-carrying cells was tracked in REL606 and M114 host strains. Populations were started directly from freezer stocks (left panels: naive) or after an initial period of evolution during which mutations that increased the fitness of plasmid-carrying cells were expected to be selected (right panels: evolved). Naive populations: *n* = 4; evolved populations: *n* = 6 (control plasmid) or 9 (*bla*_TEM-116__*_ plasmid).

To test if selected lines evolved in a way that could influence ongoing plasmid dynamics, for example, through selection of compensatory mutations that reduce purifying selection against plasmid-containing cells, we exposed them to kanamycin to select for remaining plasmid-carrying cells in each line. These populations, in which plasmid-containing cells were reset to fixation, were used to start new selection lines that were propagated through an additional 21 daily transfers. Loss of pmFP-*bla*_TEM-116*_ during this second selection period was greatly reduced in M114 lines that had been previously evolved for 400 generations compared to naive control lines in which no previous evolution had occurred (previously evolved: plasmid-containing cells at 21 days = 93.7%, 86.6–97.4 95% CI; naive: 7.9%, 3.7–15.5 95% CI; Fig. 1). In lines started with the host strain REL606, in which *bla*_TEM-116*_ conferred no detectable cost, the initial period of evolution had no effect on plasmid carriage (previously evolved: plasmid containing cells at 21 days = 60.9%, 50.6–70.3 95% CI; naive: 66%, 55.8–75.0 95% CI; Fig. 1). These results indicate that some evolutionary change occurred during the initial evolution of M114(pmFP-*bla*_TEM-116*_) lines that increased population-level maintenance of the plasmid in the absence of direct selection.

### Compensatory mutations reduced the cost of *bla*_TEM-116*_ in M114

**Fig 2:**
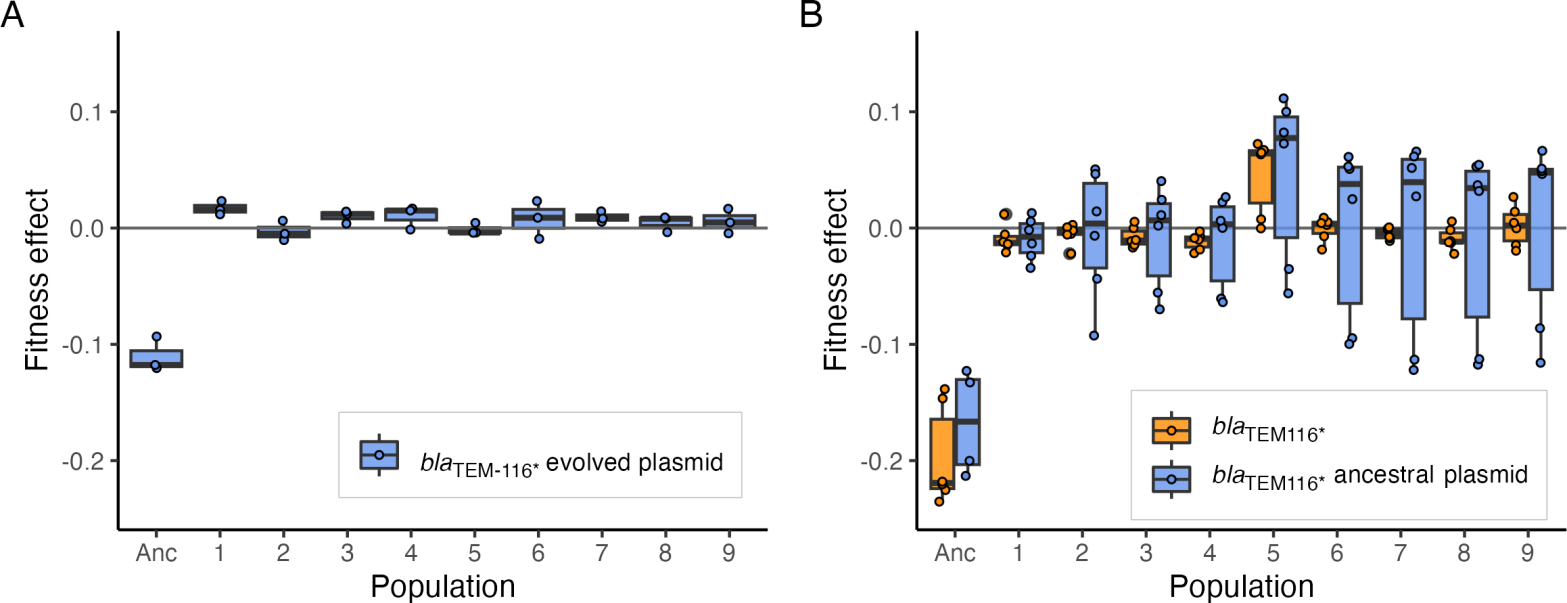
Compensation of the fitness cost of *bla*_TEM-116__*_ in evolved M114 lines. (**A**) Fitness effect of the evolved *bla*_TEM-116__*_ plasmid on the fitness of the ancestor (Anc) and clones isolated from nine independently evolved lines. Plasmid effect was estimated from the fitness difference of isolated clones and paired derivatives that were cured of the plasmid. (**B**) Fitness effect of the ancestral *bla*_TEM-116__*_ plasmid and the *bla*_TEM-116__*_ ARG in the ancestral strain (Anc) and in the same independently evolved clones shown in panel A. Plasmid effect was determined by introducing the ancestral *bla*_TEM-116__*_ plasmid into evolved clones that had been cured of the evolved plasmid. The *bla*_TEM-116__*_ ARG effect was estimated as the difference in fitness of evolved clones carrying the ancestral *bla*_TEM-116__*_ plasmid and paired clones carrying an empty vector. Boxes indicate mean and first and third quartiles, whiskers indicate up to 1.5× the interquartile range, and symbols indicate individual estimates (A, *n* = 3; B, *n* ≥ 4).

To determine if changes in pmFP-*bla*_TEM-116*_ dynamics in the previously evolved M114 lines were due to compensation of its cost, we isolated a plasmid-containing clone from each line and estimated the fitness effect of the plasmid. We found the plasmid did not impose a significant fitness effect in any evolved clone, consistent with the evolution of compensating mutations (Fig. 2A). Compensatory mutations could occur in the bacterial genome or on the pmFP-*bla*_TEM-116*_ plasmid. To distinguish between these possibilities, we cured the evolved plasmid from the isolated clones and introduced the ancestral pmFP-*bla*_TEM-116*_ plasmid into the cured clones. In no case did this plasmid confer a fitness cost, indicating that compensatory mutations reside in the bacterial chromosome (Fig. 2B). Furthermore, the fitness effects of the *bla*_TEM-116*_ ARG by itself (given by the difference in fitness of strains carrying pmFP-*bla*_TEM-116*_ or the empty pmFP vector) were not different from those of the entire pmFP-*bla*_TEM-116*_ plasmid, indicating compensation was to the ARG itself, not some other aspect of the pmFP plasmid backbone (Fig. 2B; *t*-test, *P* > 0.05 for all paired comparisons).

### Mutations on a native phage alleviate the cost of the *bla*_TEM-116*_ plasmid

To identify the genetic basis of compensatory mutations occurring in the M114 evolved clones, we sequenced the genomes of the same pmFP-*bla*_TEM-116__*_ plasmid-carrying clones in which we measured plasmid and ARG fitness effects (Fig. 2A and B). We found mutations involving a native 90 kb P1-like phage (hereafter, P1_M114_) in all clones. Six of the evolved *bla*_TEM-116__*_ plasmid clones lost the entire phage, while the other three clones gained a mutation, I179S, in a phage-encoded *relA* gene, a predicted ppGpp synthetase (Table S2) (To distinguish the phage-encoded *relA* from the chromosomal *E. coli relA* gene, we hereafter refer to it as *relA_P1_*.). By contrast, none of the six evolved control pmFP plasmid-containing lines had any mutations in P1_M114_ (Table S2). The enrichment of P1_M114_ mutations in *bla*_TEM-116__*_-expressing clones makes these changes a good candidate for underlying the parallel evolved compensation trait (Fig. 2A and B).

**Fig 3:**
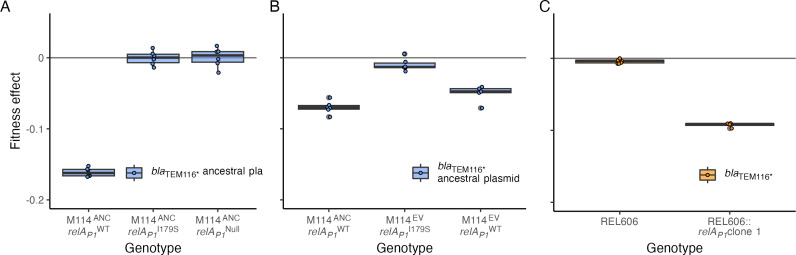
Effect of *relA_P1_* mutations on fitness effect of the *bla*_TEM-116__*_ plasmid and ARG. (**A**) Fitness effect of the pmFP-*bla*_TEM-116*_ plasmid in ancestral M114 with *relA_P1_* I179S and *relA_P1_* null alleles. (**B**) Fitness effect of the pmFP-*bla*_TEM-116*_ plasmid in the ancestral M114 strain and derivatives of an evolved M114 clone containing either the evolved *relA_P1_* I179S allele or a reverted *relA_P1_* wild-type allele. (**C**) Fitness effect of the *bla*_TEM-116*_ ARG in REL606 and a derivative encoding the ancestral *relA_P1_* gene transferred from M114. Boxes indicate mean and first and third quartiles, whiskers indicate up to 1.5× the interquartile range, and symbols indicate individual estimates (*n* ≥ 5).

To test if evolved P1_M114_ mutations are sufficient to compensate for the cost of pmFP-*bla*_TEM-116*_, we determined the plasmids’ cost in a derivative of the ancestral M114 strain containing the evolved *relA_P1_* I179S mutation (Fig. 3A). In this strain, the cost of the *bla*_TEM-116*_ plasmid was reduced from 16.1% (±0.6 95% CI) in the ancestor to being neutral (0.0% ± 1.0% 95% CI). We were unable to cure P1_M114_ from M114 cells, so we introduced a null *relA_P1_* allele into the ancestral clone to determine the effect of losing only that gene. Again, the effect of the *bla*_TEM-116*_ plasmid was changed to become neutral (0.0% ± 1.4% 95% CI; Fig. 3A).

To further characterize the genetic basis of compensation, we reverted the evolved *relA_P1_^I179S^* allele to the ancestral allele in one evolved clone. We found that this change caused the cost of pmFP-*bla*_TEM-116*_ to increase significantly (difference in mean cost 4%, *t*-test *P* < 0.001) but not to a level as great as that seen in the ancestral strain (difference in mean cost 1.9%, *t*-test *P* = 0.02) (Fig. 3B) (We note evidence of a block effect such that the effect of pmFP-*bla*_TEM-116*_ in the ancestral M114 was less in this experiment than in others reported in this study. However, competitions reported in Fig. 3B were performed in the same experimental block so that they are comparable to one another.). Together, our results indicate that evolved changes in *relA_P1_* are sufficient to remove all costs of pmFP-bla_TEM-116*_ in ancestral M114 cells but that other evolved changes also contribute some component of reducing pmFP-bla_TEM-116*_ cost in the focal evolved line.

**Fig 4:**
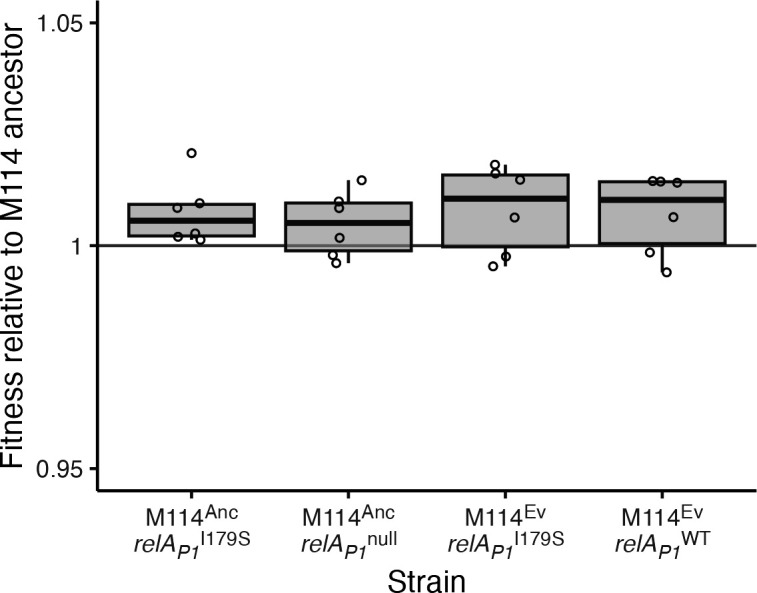
Fitness effect of *relA_P1_* in M114. Fitness of the M114 ancestor and an evolved strain with indicated *relA1_P1_* alleles relative to the M114 ancestor. In no case was a significant difference in fitness detected (*t*-test all *P* > 0.05). Boxes indicate mean and first and third quartiles, whiskers indicate up to 1.5× the interquartile range, and symbols indicate individual estimates (*n* ≥ 6).

Finally, we determined the fitness effect of *relA_P1_* alleles independent of pmFP-*bla*_TEM-116*_. We found that the I179S and null *relA_P1_* alleles had no effect on ancestral M114 fitness and that reverting the evolved *relA_P1_* I179S allele to the ancestral allele had no effect on the fitness of an evolved strain cured of the plasmid (*t*-test all *P* > 0.05; Fig. 4). Evidently, changes to *relA_P1_* affect fitness only through a positive epistatic interaction that compensates for the cost of the *bla*_TEM-116*_ ARG and do not confer any general benefit to cells in the test environment.

### Expression of *relA*_*P1*_ can cause *bla*_TEM-116*_ plasmid costs

Our results demonstrate that the wild-type *relA_P1_* gene interacts with the *bla*_TEM-116__*_ gene to cause a cost in M114. To test if the same interaction can cause *bla*_TEM-116__*_ to be costly in other genetic backgrounds, we introduced *relA_P1_* into the lab strain REL606, which does not naturally have the phage P1_M114_ or *relA_P1_* and has no cost when carrying the *bla*_TEM-116__*_ plasmid (30). If *relA_P1_* is sufficient to cause the *bla*_TEM-116__*_ plasmid to confer a cost to host cells, we expected that expressing *relA_P1_* alone would incur a cost of the plasmid similar to that in M114. By contrast, if other phage P1_M114_-encoded genes or M114-specific genes are involved, we expected that expressing *relA_P1_* alone in REL606 would have little or no influence on the fitness effect of *bla*_TEM-116__*_.

We integrated *relA_P1_* at the *att*Tn*7* site in the REL606 chromosome and measured the effect of this insertion on the cost of bla_TEM-116*_. We found that bla_TEM-116*_ confers a 9% (±0.5% 95% CI, *t*-test *P* < 0.001) fitness cost in REL606 Tn*7:: relA_P1_* but has no cost in the wild-type REL606 (cost = 0.2% ± 0.4% 95% CI, *t*-test *P* = 0.08) (Fig. 3C). In the absence of any plasmid or ARG, the *relA_P1_* insertion conferred no cost in the competition environment (*t*-test *P* = 0.16). Taken together, the compensated cost of bla_TEM-116*_ in M114 with a *relA_P1_* null mutant and the emergent cost of bla_TEM-116*_ in REL606 Tn*7:: relA_P1_* indicates that *relA_P1_* can be necessary and sufficient to cause bla_TEM-116*_ to confer a fitness cost.

### Similarity of *relA*_*P1*_ to chromosomal relA/spoT and prevalence in phage

RelA_P1_ shares sequence similarity to the N-terminal enzymatic domains of the *E. coli* ppGpp synthetase RelA and ppGpp synthetase/hydrolase SpoT but lacks their C-terminal regulatory domains (Fig. S1). Sequence alignment of RelA_P1_ to RelA and SpoT showed that the mutated I179 residue of RelA_P1_ is located near residues critical for ppGpp synthetase activity, suggesting both that RelA_P1_ has functional ppGpp synthetase activity and that this activity is affected by the evolved mutation (Fig. S1). We note that the null and I179S *relA_P1_* alleles caused a similar reduction in the cost of the *bla*_TEM-116*_ plasmid consistent with the I179S substitution causing a loss of RelA_P1_ activity (Fig. 3B). It is unclear why the same loss-of-function mutation would occur independently in three lines.

**Fig 5:**
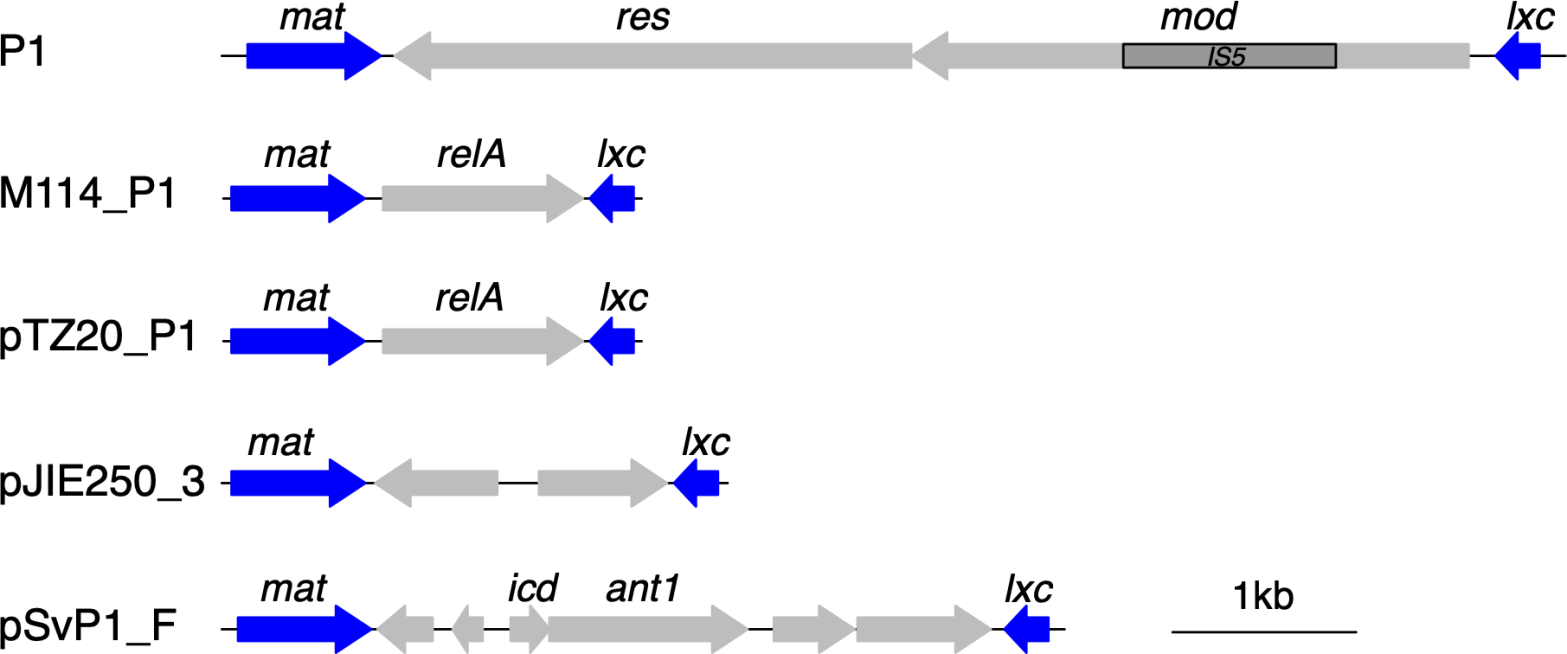
Synteny of the P1 RD-1 region shows a variable gene repertoire. A close-up of the RD-1 of phage P1 (from Fig. S2). The RD-1 region is consistently flanked by *mat* and *lxc* genes, but internal gene content is variable [unnamed genes encode putative transcription factors or proteins of unknown function (43)]. The bottom three rows correspond to elements designated as P1-like plasmids (40).

The *relA_P1_* gene is not found in all P1 phages (Fig. 5 and S2). In isolates that do encode *relA_P1_*, it is found in a variable and comparatively low GC area designated as a region of difference 1 (RD-1) that is thought to comprise recently acquired genes (40). To survey the incidence of *relA_P1_* in P1 phages, we used the web-based BLASTn platform with default settings to first identify sequence accessions with two characteristic features of P1 phage, the phage replication protein, *repL*, and the P1 plasmid replication protein, *repA* (40, 41). Using a cutoff of 90% query coverage and sequence identity, 141 accessions matched both features. Of these, six also matched *relA_P1_*. We also found matches to *relA_P1_* in 25 additional accessions. In all cases, *relA_P1_* matches were flanked by the same *mat* and *lxc* genes found in the M114 P1 element (Fig. S2 and S5). These genes are involved in phage-related processes—*mat* in phage maturation and *lxc* as a modulator of C1-mediated repression—consistent with most matches occurring in phage or phage-like elements, even if they do not encode *repL* and *repA* (42). The strong genetic linkage between *relA_P1_* and flanking genes occurring in an otherwise variable region suggests a recent spread of *relA_P1_* among a range of phages.

## DISCUSSION

We investigated the genetic basis of a host-dependent cost of *bla*_TEM-116*_ and of compensation to this cost. This ARG was previously demonstrated to impose a significant fitness cost to one *E. coli* host strain, M114, but to have little effect in 10 other tested strains (30). We found that changes to a P1-like bacteriophage element present in M114, resulting in either loss of the element or loss of function of *relA_P1_*, a gene it encoded, occurred in all nine evolved clones that compensated the cost of *bla*_TEM-116*_. Genetic manipulation experiments demonstrated that the evolved *relA_P1_* mutation was necessary and sufficient to account for compensation of the cost of *bla*_TEM-116*_ in M114. Moreover, the introduction of *relA_P1_* in a naive host, REL606, in which *bla*_TEM-116*_ was initially neutral, caused *bla*_TEM-116*_ to confer a significant cost. These results demonstrate that the cost of *bla*_TEM-116*_ seen in this study was the result of an antagonistic interaction between phage-encoded *relA_P1_* and *bla*_TEM-116*_, highlighting the potential influence of bacterial accessory genomes on the maintenance and evolution of ARGs.

Several mechanisms of β-lactamase cost have been identified. For example, the costs of BlaCTX-M-15, BlaSME-1, BlaVIM-1, and BlaSPM-1 depend on their signal peptides, which are required to translocate the β-lactamases across the inner membrane to the periplasm and can increase cell envelope stress (17–19). Other β-lactamases have low levels of DD-transpeptidase activity, which may impose costs by affecting normal peptidoglycan synthesis (20). We did not determine the mechanistic basis of *bla*_TEM-116*_ cost in the M114 strain, but we did demonstrate that its cost depends on the presence of the phage-encoded *relA_P1_*. Protein sequence alignments indicate that RelA_P1_ has a similarity to the ppGpp synthetase domain of the chromosomal RelA and SpoT enzymes and that it lacks any recognized regulatory domain (Fig. S1). During nutrient starvation, *E. coli* ppGpp levels increase sharply via the activation of RelA or SpoT ppGpp synthetase activity (44). ppGpp regulates various genes involved in cell physiology, resulting in growth arrest as part of a so-called stringent response. In the absence of any regulatory domain, the phage-encoded RelA_P1_ may increase the basal level of ppGpp and hypersensitize cells to mild stresses. Hypersensitization could lead to a fitness cost in the presence of BlaTEM-116* if that protein compromised the cell envelope or affected peptidoglycan structure. These changes might be tolerated in the wild-type cells but induce a stringent response in RelA_P1_-hypersensitized cells (45).

The function of *relA_P1_* in the context of the phage P1_M114_ is unclear. A candidate adaptive mechanism for RelA_P1_ functioning as a small ppGpp synthetase enzyme is as a toxin or component of a toxin-antitoxin module (46, 47). For example, a homolog of ppGpp synthase, Tas1, which pyrophosphorylates adenosine nucleotides to produce ppApp, can be delivered via a type VI secretion system to neighboring cells to cause cell death (48). However, we found that *relA_P1_* expression did not decrease cell fitness, suggesting either that RelA_P1_ is not a toxin or that the hosts already encoded a cognate antitoxin. Alternatively, if RelA_P1_ increases basal ppGpp levels, lowering the threshold for stringent response induction, an encoding phage might gain a selective advantage by early induction of the lytic cycle in harsh environments or through abortive infection providing population-level resistance to secondary infection (43). Moreover, the elevated basal level of ppGpp may increase the proportion of persister cells induced by stochastic activation of the stringent response (49). Increased spontaneous persister cells may increase the survival of the host population (and hence the temperate phage) in fluctuating environments.

Whatever the basis of the genetic interaction between *relA_P1_* and *bla*_TEM-116*_, it is exclusive to that β-lactamase among a set of three that we have considered. In the previous work, we found that two other β-lactamases, BlaCTX-M-15 and BlaSHV12, did not confer significant costs when introduced into M114. We also note that one sequenced P1 strain, pTZ20-1P, encodes both *relA_P1_* and *bla*_TEM-1_ (Fig. S2) (40). It would be interesting to investigate if the antagonism between these genes exists in this phage and if not, how it has been resolved. In any case, studying the interaction dynamics of *relA_P1_* and *bla*_TEM-116*_ may have practical application in developing approaches to reduce the dissemination of *bla*_TEM-116*_ by increasing its cost to host cells.

Models can provide important insight into the implications of strain-specific fitness effects for the ecological dynamics of ARG-encoding plasmids. A study that examined the effect of a carbapenemase-carrying plasmid, pOXA-48_K8, over a set of 50 enterobacterial isolates found large differences in its effect on fitness, ranging from a ~20% benefit to a ~ 20% cost across a set of enterobacterial isolates (50). Such variation was predicted to be beneficial for the maintenance of the plasmid in a complex bacterial community, allowing it to be maintained in strains in which it confers a low cost in environments not containing the corresponding antibiotic, then being transferred and selected in higher-cost strains in environments containing the antibiotic (50).

Specific predictions of ARG-plasmid dynamics are, however, complicated by the potential for compensatory mutations to change fitness effects over time (21–26). Even the location—on the host chromosome or on the costly plasmid itself—of compensatory mutations, let alone the effect and the rate at which they occur, has been shown to have a major impact on success (51). Our demonstration that both the cost of an ARG and the subsequent compensation of that cost can depend on a secondary horizontally mobile element (HME) represents a further complication. If costs and compensation depend on the presence and success of a secondary HME, changes in ARG costs can not only change quickly and independently of host genotype characteristics but may also be selected unpredictably depending on factors that favor the spread of the HME. Nevertheless, we note that there remain biases in the presence of ARGs in different species—for example, the metallo-β-lactamases BlaVIM-2 and BlaSPM-1 are frequently found in *Pseudomonas aeruginosa* but rarely in enterobacteria, likely due to differences in their costs—suggesting that not all costs can be overcome (19).

In summary, we demonstrate a rapid selection of host cells to compensate for the cost of the *bla*_TEM-116*_ resistance determinant. Compensation mutations included deletion or point mutations in a phage-encoded gene, *relA_P1_*, which we demonstrate interacts antagonistically with *bla*_TEM-116*_. It is unclear if such antagonistic interactions are common, but a survey on the fitness cost of ~200 ARGs has shown that cell-interacting ARGs are more likely to incur a cost (15), emphasizing the importance of the compatibility between a HGT gene and its host. Future studies on the identification of the genetic antagonism between an ARG and its host and on the significance of the antagonistic interactions on the dissemination and maintenance of ARGs in bacterial populations will contribute to a comprehensive view on the flow of ARGs between bacteria in nature.

## Data Availability

T.F.C. will make the strains constructed in this study available to qualified recipients following the completion of an institutional material transfer agreement. The results of the competition experiments, summary input data, and analysis scripts that pertain to the experiments and analyses reported in this paper have been deposited at Dryad.

## SUPPLEMENTAL MATERIAL

Supplemental material mbio.02776-23-s0001.pdf Supplemental tables and figures.

ASM does not own the copyrights to Supplemental Material that may be linked to, or accessed through, an article. The authors have granted ASM a non-exclusive, world-wide license to publish the Supplemental Material files. Please contact the corresponding author directly for reuse.
