# Supplementary material for: Interaction with a phage gene underlie costs of a β-lactamase
Source: mBio. 2024 Jan 9;15(2):e02776-23. doi: 10.1128/mbio.02776-23 (PMC10865808; doi:10.1128/mbio.02776-23)
Supplement: Supplemental material — Supplemental tables and figures. [file mbio.02776-23-s0001.pdf]

**Supplementary information for:**

# Interactions with a phage gene underlie costs of a β-lactamase

Huei-Yi Lai[1] and Tim F. Cooper[1]

[1] School of Natural Sciences, Massey University, Auckland 0634, New Zealand

**Table S1. Oligonucleotides used to introduce mutations into the P1-like phage gene *relA*$_{P1}$**

| Oligo ID | Purpose | Sequence (5' – 3') |
|---|---|---|
| MAGE_relA_anc_o1 | Revert evolved *relA*$_{P1}$$^{I179S}$ allele | C*T*TCGATCAGAAAGAGGATCAATATCCGTGGCGAAAAACAAAGATTGAGATACAGTTAAGAACTCAATTGCAGCATGCTTGGGCTACTAG |
| MAGE_relA_null_o1 | Introduce null *relA*$_{P1}$ allele (incorporating consecutive stop codons) | C*T*TCGATCAGAAAGAGGATCAATATCCGTGGCGAtAAACAtAGATTtAGATAtAGTTAAGAACTCAATTGCAGCATGCTTGGGCTACTAG |
| MAGE_relA_evo_o1 | Introduce evolved *relA*$_{P1}$$^{I179S}$ allele | C*T*TCGATCAGAAAGAGGATCAATATCCGTGGCGAAAAACAAAGAgTGAGATACAGTTAAGAACTCAATTGCAGCATGCTTGGGCTACTAG |

* phosphorothioated nucleotides

Lower-case nucleotides indicate changes relative to the ancestral allele.

Table S2. Mutations identified in evolved plasmid-carrying clones.

| Control (pmFP) vector lines | | | | | | pmFP-*bla*$_{TEM\text{-}116*}$ lines | | | | | | | | | Gene | Description |
|---|---|---|---|---|---|---|---|---|---|---|---|---|---|---|---|---|
| 1 | 2 | 3 | 4 | 5 | 6 | 1 | 2 | 3 | 4 | 5 | 6 | 7 | 8 | 9 | | |
| Δ1bp | Δ72bp | | | Δ1bp | | | | | | | | | | | *GIAEAHOA_00676* | O-antigen polymerase |
| | | | K36* | | | | | | | | | | | | *rfaL* | O-antigen ligase |
| | | P52S | | | | | | | | | | | | | *dut* | dUTPase |
| R562C | E82* | R62L | | R562C | R562C | | | | L92Q | Q280K | | | | +1bp | *prkA* | Serine protein kinase |
| | | | | | | | | | | | | A196V | | | *ydhP* | MFS transporter |
| | | | | | | Q407* | | | | | | | S318P | | *yhbH* | Probable sigma(54) modulation protein |
| | | | | | | | | | | +2bp | | | | | *GIAEAHOA_00573* | Phage tail protein |
| | | | | | | | H210Y | | | | | | | | *GIAEAHOA_02332* | Glycosyltransferase |
| | | | | | | | | | | | | | D58Y | | *nfsA* | Nitroreductase |
| | | | | | | | | | | | D54bp | | | | *ycgB* | Putative SpoVR family protein |
| | | | | | | Loss | Loss | | Loss | Loss | Loss | | | Loss | *Phage P1_M114* | Phage P1_M114 |
| | | | | | | | | I179S | | | | I179S | I179S | | *relAP1* | Putative small ppGpp synthase |

* Intergenic and synonymous mutations are excluded from this table.

** Mutations affecting the *relAP1* gene are highlighted in the box at the bottom of the table for emphasis.

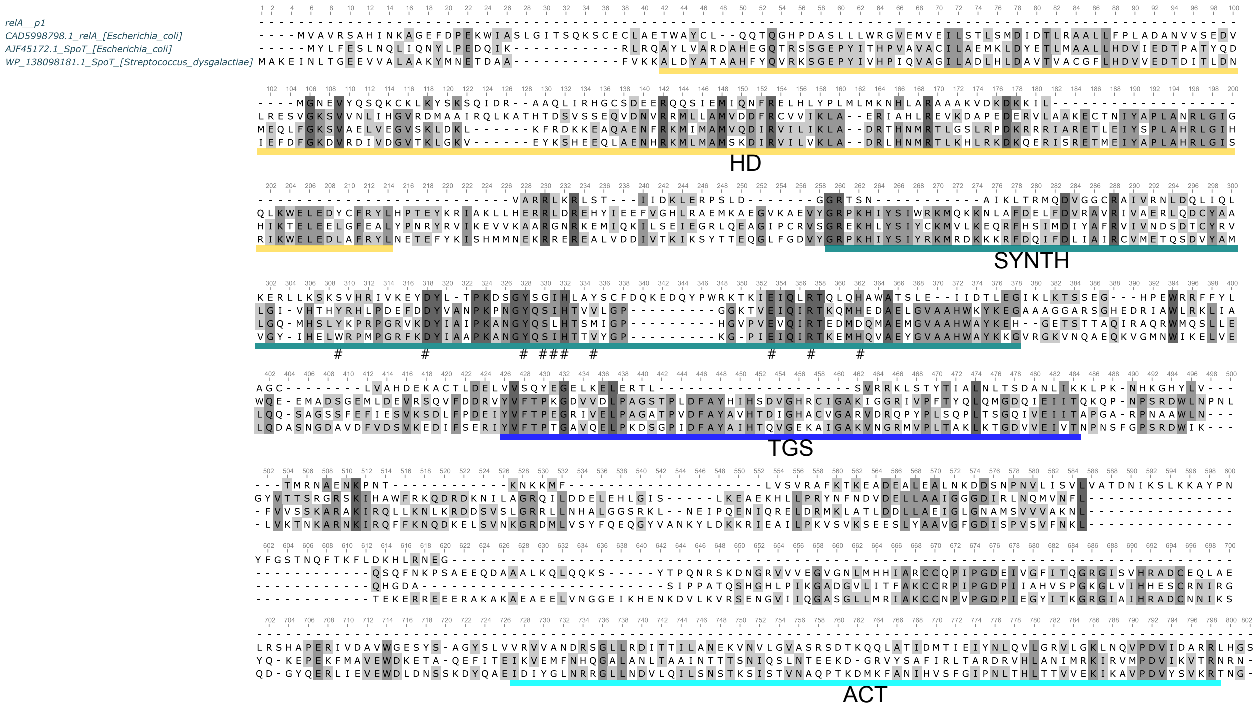


**Figure S1 Protein alignment of RelA/SpoT homologs.** Protein homologs of RelA/SpoT from different species origins were aligned with CLUSTALW 2.0. SpoT from *Streptococcus dysgalactiae* is a bifunctional protein with both ppGpp synthetase (SYNTH, green line) and hydrolase (HD, yellow line) activity in the N-terminal domain. RelA from *E. coli* only has the synthetase activity, and SpoT from *E. coli* has both synthetase and hydrolase activity. The C-terminal region of the full length RelA/SpoT proteins contains regulatory domains (TGS, blue line, and ACT, cyan line) that binds to other elements such as ribosomes. $RelA_{P1}$ from phage P1 aligns to the N-terminal domain of the full length RelA/SpoT. The $RelA_{P1}$-I179S mutation is at position 352 on this alignment. Amino acids critical for the synthetase activity around I179 of $RelA_{P1}$ are marked with the hash symbol. Protein domains and amino acid sites necessary for enzymatic activity were manually added in the alignment based on published data (Hogg et al. 2004; Atkinson et al. 2011).

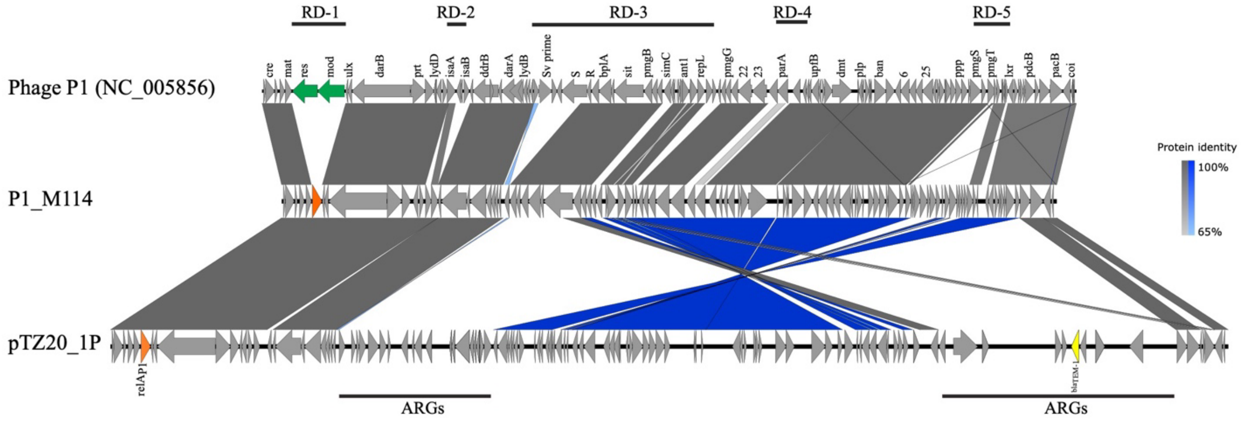


**Figure S2. Genetic map and alignment of phage P1 variants.** Genome comparison between phage P1 variants. Regions noted as RD1-5 indicate areas of sequence difference between a range of P1 isolates (Venturini et al. 2019). The protein sequence identity between P1$_{M114}$ and either pTZ20_1P or the canonical lab strain (NC_005856) is colored by grey or blue (inverted region) color gradients. The *relA*$_{P1}$ gene (orange arrow) is present in P1_M114 and pTZ20_1P but absent in the corresponding region of the lab strain phage (alternative genes are identified with green arrows). pTZ20_1P has acquired multiple ARGs, including *bla*$_{TEM-1}$ (yellow arrow). Genome comparison and visualization of the P1 variants were done using Easyfig (Sullivan et al. 2011).

## References


Atkinson GC, Tenson T, Hauryliuk V. 2011. The RelA/SpoT homolog (RSH) superfamily: Distribution and functional evolution of ppGpp synthetases and hydrolases across the tree of life. *PLoS ONE* 6:e23479.

Hogg T, Mechold U, Malke H, Cashel M, Hilgenfeld R. 2004. Conformational antagonism between opposing active sites in a bifunctional RelA/SpoT homolog modulates (p)ppGpp metabolism during the stringent response. *Cell* 117:57–68.

Sullivan MJ, Petty NK, Beatson SA. 2011. Easyfig: a genome comparison visualizer. *Bioinformatics* 27: 1009-1010.

Venturini C, Zingali T, Wyrsch ER, Bowring B, Iredell J, Partridge SR, Djordjevic SP. 2019. Diversity of P1 phage-like elements in multidrug resistant Escherichia coli. *Sci. Rep.* 9:18861.
